# A Snapshot on the Genomic Epidemiology of Turkey Reovirus Infections, Hungary

**DOI:** 10.3390/ani13223504

**Published:** 2023-11-13

**Authors:** Bence Gál, Renáta Varga-Kugler, Katalin Ihász, Eszter Kaszab, Szilvia Farkas, Szilvia Marton, Vito Martella, Krisztián Bányai

**Affiliations:** 1 Intervet Hungária Kft, Lechner Odon Fasor 10/b, H-1095 Budapest, Hungary; bence.gal@merck.com; 2 Veterinary Medical Research Institute, Hungária krt. 21, H-1143 Budapest, Hungary; kugler.renata@vmri.hun-ren.hu (R.V.-K.); ihasz.katalin@vmri.hun-ren.hu (K.I.); kaszab.eszter@vmri.hun-ren.hu (E.K.); marton.szilvia@vmri.hun-ren.hu (S.M.); 3 National Laboratory for Infectious Animal Diseases, Antimicrobial Resistance, Veterinary Public Health and Food Chain Safety, Hungária krt. 21, H-1143 Budapest, Hungary; 4 Institute of Metagenomics, University of Debrecen, Nagyerdei krt. 98, H-4032 Debrecen, Hungary; 5 Department of Obstetrics and Food Animal Medicine Clinic, University of Veterinary Medicine, István utca 2, H-1078 Budapest, Hungary; farkas.szilvia@univet.hu; 6 Department of Veterinary Medicine, University of Bari, Aldo Moro, S.P. per Casamassima km 3, 70010 Valenzano, Italy; vito.martella@uniba.it; 7 Department of Pharmacology and Toxicology, University of Veterinary Medicine, István utca 2, H-1078 Budapest, Hungary

**Keywords:** *Avian orthoreovirus*, *Spinareoviridae*, whole-genome sequencing, phylogenetic analysis, reassortment, interspecies transmission

## Abstract

**Simple Summary:**

A genomic epidemiological study was conducted to describe the genetic diversity within turkey reoviruses circulating in Hungary. Phylogenetic analyses confirmed that turkey reoviruses comprise a well determined genetic clade; however, evidence shows occasional genetic interaction between turkey reoviruses and reoviruses of other avian species. A better understanding of the source and the route of reovirus infections in the field requires extended sampling of strains from additional geographic areas and from different phases of turkey production.

**Abstract:**

Reovirus infections in turkeys are associated with arthritis and lameness. Viral genome sequence data are scarce, which makes an accurate description of the viral evolution and epidemiology difficult. In this study, we isolated and characterized turkey reoviruses from Hungary. The isolates were identified in 2016; these isolates were compared with earlier Hungarian turkey reovirus strains and turkey reoviruses isolated in the 2010s in the United States. Gene-wise sequence and phylogenetic analyses identified the cell-receptor binding protein and the main neutralization antigen, σC, to be the most conserved. The most genetically diverse gene was another surface antigen coding gene, μB. This gene was shown to undergo frequent reassortment among chicken and turkey origin reoviruses. Additional reassortment events were found primarily within members of the homologous turkey reovirus clade. Our data showed evidence for low variability among strains isolated from independent outbreaks, a finding that suggests a common source of turkey reoviruses in Hungarian turkey flocks. Given that commercial vaccines are not available, identification of the source of these founder virus strains would permit a more efficient prevention of disease outbreaks before young birds are settled to fattening facilities.

## 1. Introduction

Turkey reovirus (TRV) infections have been associated with diverse diseases in their homologous hosts, the common turkey (*Meleagris gallopavo*). Turkey arthritis is characterized by lameness, and uneven growth is commonly seen in affected flocks. When internal organs are involved, hepatitis, myocardial or intestinal manifestations are also reported [1,2,3,4,5]. Immune suppression due to atrophy of the bursa *Fabricii* and the thymus has also been documented [6,7]. The increased susceptibility to secondary infections of immune-compromised birds may complicate the reovirus-associated pathology and worsen the disease outcome. The global economic impact of TRV arthritis is not fully known but estimates from the United States suggest a national loss of ~USD 33 million per year [2].

The species *Avian orthoreovirus* is a member of the genus *Orthoreovirus*, family *Spinareoviridae* [8]. This orthoreovirus species comprises the vast majority of strains isolated from domestic birds (such as ducks, geese, chicken, turkey) and reared gamebirds (including pheasant and partridge) [9,10]. The viral genome consists of 10 segments of double-stranded RNA, which are classified based on migration in polyacrylamide gel as L (large, L1 to L3), M (medium, M1 to M3) and S (small, S1 to S4) class genome segments. The size of the complete genome of turkey origin orthoreovirus strains is in the range of 24 kilo base pair. Typically, the genome encodes 12 proteins, three on L (λA, λB and λC), three on M (μA, μB and μNS) and six on S class genome segments (p10, p17, σC, σA, σB and σNS). The S1 genome segment is tricistronic (encoding p10, p17 and σC) with partially overlapping genes [11].

Avian orthoreoviruses naturally undergo various evolutionary mechanisms. These processes include genetic drift associated with the accumulation of point mutations and exchange of genetic information between homologous genome segments through reassortment or recombination [9,12]. The two latter mechanisms require co-infection of a susceptible cell with different strains to potentially record phenotypic alterations. Reassortment generates novel constellations of the 10 genomic segments and is very common among homologous orthoreovirus strains. Viral host-switching with or without reassortment may also occur.

Although TRVs share genomic and phylogenetic similarities with chicken and other galliform bird origin reoviruses, in most instances, they can be distinguished by phylogenetic analyses [13,14,15]. The genetic relationship among TRVs from the Americas and Europe has revealed a very close relationship in past studies [13,16]. In this study, we sequenced eight TRVs isolated from two Hungarian turkey flocks and performed gene-by-gene phylogenies in comparison with orthoreoviruses isolated from other galliform birds. The aim of the study was to better understand the origin and inter-strain variability of TRVs that circulated in different periods.

## 2. Materials and Methods

### 2.1. Cases

Reovirus infections in turkeys were diagnosed in samples originating from three geographic locations in Hungary (Békés, Vas and Veszprém counties, respectively) during 2016. Location #1 (in Vas county) included samples that were collected at a slaughterhouse dedicated, in general, to processing poultry meat but restricted to processing exclusively turkey in the sampling period. Location #2 (in Veszprém county) was a poultry farm with two flocks of 14-days-old male turkeys. Roughly 10% of animals were affected by lameness and uneven growth rate. No increased mortality was recorded but lame birds died over time as they were unable to access food. Upon necropsy, serous-fibrinous hemorrhagic inflammation of the tarsometatarsal joint was recorded. No other clinical signs or pathological alterations were seen. Location #3 (in Békés county) was a fattening flock with 8-weeks-old birds. The observed clinical signs were lameness and uneven growth rate that affected 20% of the flock and affected both sexes. Necropsy uncovered inflammation of the tarsometatarsal joint, but no other alterations were observed.

### 2.2. Specimens

A total of 12 intestine and 20 tendon specimens were collected from dead birds (*n* = 32) from the three locations (Vas county, 7 intestines; Veszprém county, 10 tendons; Békés county, 10 tendons and 5 intestines). Laboratory investigation included virus isolation followed by virus-specific RT-PCR and sequencing of σC gene from samples that produced cytopathogenic effect (CPE) in cell culture. Pathogens other than reoviruses were not sought in the samples.

### 2.3. Virus Isolation and Identification

For virus isolation, the gut and tendon specimens were homogenized individually in sterile PBS solution. The supernatants of centrifuged homogenates were filtered using 0.45 µm PES syringe filters and then inoculated on chicken hepatocellular carcinoma cell line LMH (Leghorn male hepatoma, ATCC CRL-2117) in the presence of DMEM (Lonza), 5% fetal bovine serum (Gibco) and 1% antibiotic/antimycotic solution (Lonza). Virus culturing was carried out at 37 °C with a constant supply of 5% carbon dioxide. Giant cell formation, as typical cytopathogenic effect (CPE) of TRVs, was the primary endpoint of virus isolation. When CPE was not seen following 5–6 days post-inoculation, three blind passages were carried out [17].

Virus isolation results were confirmed by RT-PCR amplification and sequencing of the σC-coding gene when CPE was seen. In brief, cell culture supernatants were used to extract viral RNA (Direct-zol RNA Miniprep; Zymo Research). A home-made avian reovirus σC gene-specific RT-PCR assay using the QIAGEN One-Step RT-PCR Kit was carried out with published primers (S1_all_F3 (Fw) 5′-GATACTSTCNTTGACTTCGA-3′ and S1_all_R2 (Rev) 5′-TCGATGCCSGTACGCACGGT-3′) to obtain a ~900 nucleotide (nt) long fragment of the S1 genome segment [17]. Gel-extracted bands were subjected to Sanger sequencing with the BigDye Terminator v3.1 Cycle Sequencing Kit. The sequence chromatograms were generated on an ABI PRISM^®^ 3100-Avant Genetic Analyzer (Delta Bio 2000 Ltd., Gödöllő, Hungary).

### 2.4. Whole-Genome Sequencing

A total of eight (out of nine, 89%) TRV isolates were subjected to whole-genome sequencing. In brief, random primed RT-PCR was performed to generate cDNA from viral RNA. The DNA was purified and then measured with Qubit 2.0 equipment using the Qubit dsDNA BR Assay Kit (Thermo Scientific, Waltham, MA, USA). The Illumina^®^ Nextera XT DNA Library Preparation Kit (Illumina, San Diego, CA, USA) and the Nextera XT Index Kit v2 Set A (Illumina, San Diego, CA, USA) were used to prepare Illumina specific libraries from the random PCR product. Libraries were pooled and, at a final concentration of 1.5 pM, were loaded onto a NextSeq 500/550 Mid Output flow cell and sequenced using an Illumina^®^ NextSeq 500 sequencer (Illumina, San Diego, CA, USA) [18].

### 2.5. Data Analysis

The chromatograms of Sanger sequencing generated from the σC gene were assembled and edited using BioEdit and AliView [19,20]. Illumina sequence reads from the shotgun library DNAs were assembled using the Geneious Prime software, version 2022.2.2 [21]. BLASTn and BLASTx algorithms were used to identify homologous genes among sequences deposited in GenBank [22]. Codon-based multiple sequence alignment was generated using the Muscle algorithm within the Geneious Prime software [21]. Phylogenetic analysis was performed, and sequence identity values were calculated using the MEGAX package [23]. Sequence identity values were calculated using the *p*-distance method. The gene-specific substitution model was evaluated, and the best-fit model was selected based on the Bayesian information criterion. The maximum-likelihood trees were generated, and tree topologies were validated by bootstrap (500) analysis as implemented in MEGAX. Diversity analysis was conducted using the Maximum Composite Likelihood model as implemented in the MEGA11 software [24]. The violin and line plots were generated using OriginPro (OriginLab Corporation, Northampton, MA, USA, version 2021).

Reference avian reovirus sequences were downloaded from GenBank. Selection criteria included the host origin and the location of strains. In fact, we downloaded all sequence records that indicated that the strain was isolated from turkey, and we downloaded all reovirus sequences originating from galliform birds that were isolated from Hungary. The suitability and inclusion of sequences in our analyses depended on the length and quality of sequences. In addition, reovirus gene sequences were re-used from our previous analyses if they carried relevant information for the current study.

Viral isolates were classified based on the σC protein as recommended by Kant and co-workers [25].

## 3. Results

### 3.1. Classification Based on σC

Two intestine and seven tendon specimens contained a CPE-producing agent on LMH cells. Although turkeys with successful virus isolation originated from all three locations (one isolate from Vas county, six isolates from Veszprém county, two isolates from Békés county), only isolates from clinical cases were further analyzed. The single isolate from the slaughterhouse (located in Vas county) was not characterized due to possible contamination from other avian species processed at the slaughterhouse shortly before the sampling period. The remaining eight isolates tested positive by RT-PCR, yielding visible amplicons of ~900 nt in length, which corresponded to the expected size.

**Figure 1:**
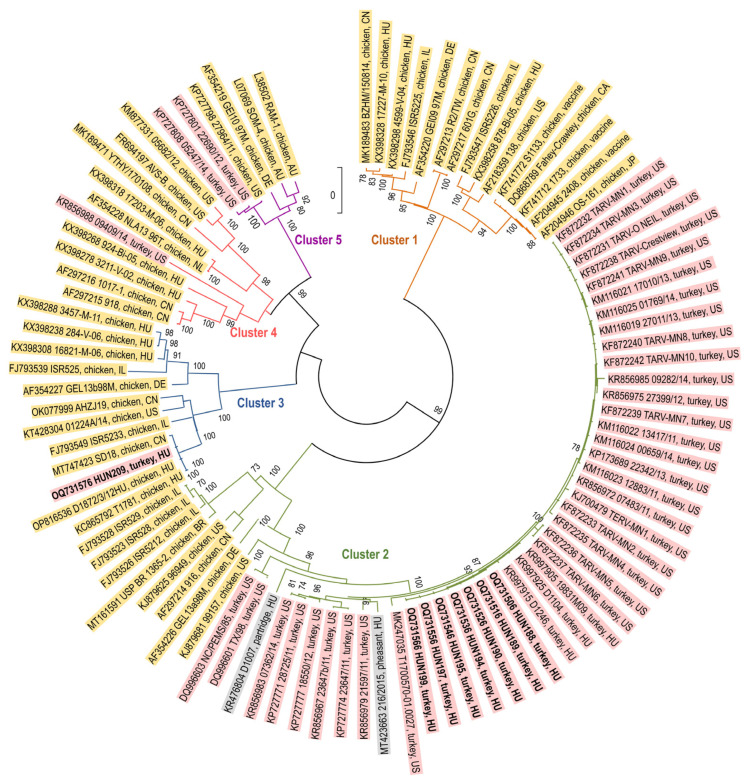
Unrooted phylogenetic tree based on the σC protein. Strain labels are colored by host species: pink, turkey; yellow, chicken; grey, pheasant and partridge. Genotypes are marked by the coloring of the tree branches (based on Kant et al. [25]).

The amplicons of the σC gene were analyzed to classify the isolates. This analysis revealed that seven isolates belonged to cluster 2 (six from Veszprém, one from Békés) while a single isolate from Békés county belonged to cluster 3 (Figure 1). Of interest, the seven strains within cluster 2 were all isolated from tendons, while the single cluster 3 strain was isolated from a gut sample. Additionally, the cluster 2 TRVs shared 100% nt identity among each other and >99% nt identity with 2009 TRV strains from Hungary, 95.8 to 97.2% nt identities with cluster 2 TRVs from the United States and up to 84% and 93.5% nt identities with Hungarian partridge and pheasant origin cluster 2 RVs, respectively. The nucleotide similarity with chicken origin cluster 2 RVs ranged from 65.6% to 78.76%. Some US origin TRVs were classified into clusters 4 and 5 where typically chicken origin RVs belonged to.

### 3.2. Genome and Gene-Wise Analyses

The genomic organization of all Hungarian study strains was similar and corresponded with that of other reference TRVs, including former Hungarian TRV isolates and TRVs reported from the USA [14,16,26,27,28]. The full-length genomes were 23,498 bp in seven study strains and 23,494 bp long in a single strain. In the latter isolate, sequence length differences were seen in some of the 5′ UTRs (untranslated regions) and the p17 protein coding gene. Despite these small differences, all TRVs were found to encode 12 primary gene products (λA, 1293 amino acids (aa) in length; λB, 1259 aa; λC, 1285 aa; µA, 732 aa; µB, 676 aa; µNS, 635 aa; σA, 416 aa; σB, 367 aa; σC, 326 aa; σNS, 367 aa; p10, 99 aa; and p17, 150 aa in seven strains and 146 aa in a single strain).

**Figure 2:**
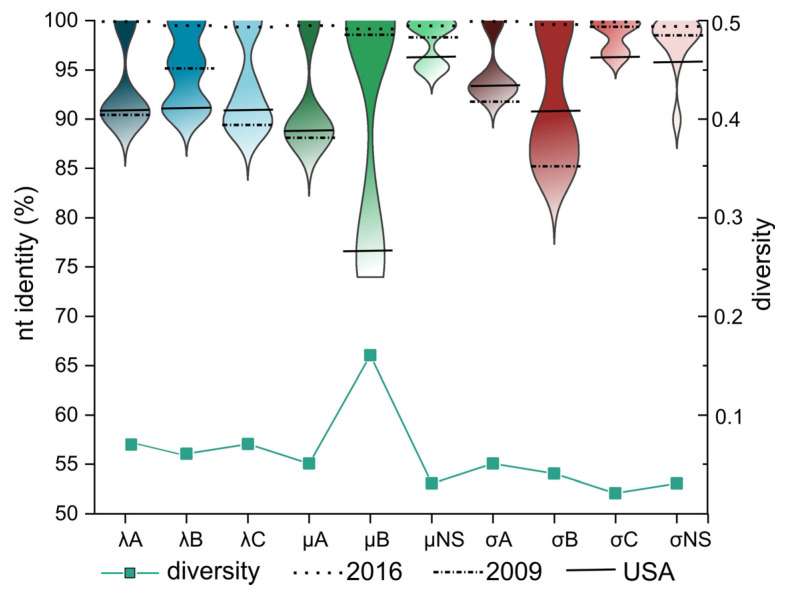
Range of sequence identities and evolutionary diversity within cluster 2 TRVs. Violin plots represent the range of nt identity values and the relative frequency of any nt identity value within the range. Lines in the violin plots show the mean sequence identity values between 2016 Hungarian TRVs (dotted line), the mean sequence identity values between the 2016 and the 2009 Hungarian TRVs (dot-dashed line) as well as the mean sequence identity values between the 2016 Hungarian TRVs and the US TRVs (solid line). The line plot represents the estimation of the mean evolutionary diversity for the entire population; the number of base substitutions per site from mean diversity calculations for the entire population are shown.

Seven TRV strains (six from Veszprém, one from Békés) shared nearly complete nt sequence identity with each other along the full genome. The number of single nucleotide variations (SNVs; in total, 13) varied by strains and genes (none in genome segments coding for λA, σA and σC and up to four SNVs in the remainders). About half (~53.8%) of the mutations were synonymous mutations (Table 1). Additionally, these seven 2016 TRV strains shared high gene-wise sequence identities with Hungarian TRV isolates from 2009 and all US strains with available genome sequence data reported from years 2010 to 2017 (Figure 2). The sequence identities ranged between 84.7% and 99.4% when comparing 2009 and 2016 strains from Hungary, with μB, μNS, σC and σNS genes being the most conserved and the σB gene the least conserved. Similar ranges of sequence similarities were obtained between the 2016 year Hungarian and that of the US cluster 2 TRVs (range, as low as 74.7% for μB and as high as 97.2% for σC). Surprisingly, when analyzing the whole data set for cluster 2 TRVs, the lowest sequence diversity was obtained for the neutralization antigen coding gene, σC, followed by μNS and σNS genes. The greatest diversity was obtained for the μB gene.

A single study strain, HUN209, from location #3 (Békés county) was genetically related to chicken origin reoviruses and particularly to a 2012 Hungarian chicken isolate, T1781. This unusual turkey isolate shared a maximum of 83.1% and 83.7% gene-wise identity (both seen in λA) with common TRV isolates reported from Hungary and the United States, respectively. The lowest sequence identities were obtained for the σC gene in these comparisons (range, 50.6% to 54.3%).

**Figure 3:**
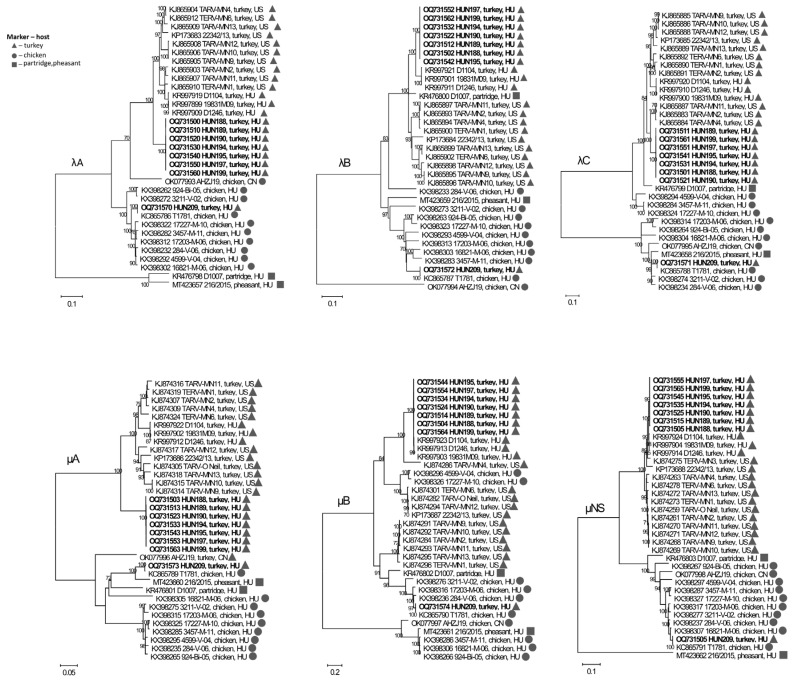
Unrooted phylogenetic trees based on the nucleotide sequences of the L- and M-class genes of different ARVs. Markers indicate host species: triangle, turkey; circle, chicken; square, pheasant/partridge. The scale bar is proportional to the genetic distance. Bootstrap values greater than 60 are shown at the branch nodes.

Phylogenetic analyses echoed the sequence similarity data, highlighting multi-gene reassortment events during comparison, e.g., of the Hungarian TRVs from 2016 and 2009 with each other. When analyzing the L-class genes, the λA phylogeny revealed that the 2016 Hungarian strains form a separate cluster while the 2009 Hungarian TRVs cluster with all currently known US TRVs (Figure 3). In contrast, the λB phylogeny uncovered close genetic relationship between 2016 and 2009 Hungarian strains, and the cluster of these strains clearly separated from the US strains. Of interest, a Hungarian partridge reovirus also clustered with TRVs in the λB phylogeny. A third pattern of topology was seen when analyzing the λC coding gene; this showed that all 2016 Hungarian isolates cluster with a set of US strains (such as MN2, MN4 and MN11), while all 2009 Hungarian strains form a shared cluster with the rest of the US strains.

Concerning the M-class genes, μA shared the topology with λA when focusing on TRVs exclusively (Figure 3). In the μB gene phylogeny, all Hungarian strains formed a common cluster with a single US strain, MN4. All other US TRVs seemed to be more closely related to chicken origin RVs, even if this selection contained only Hungarian chicken origin orthoreovirus strains and a Hungarian partridge reovirus. This figure is consistent with the multiple reassortment event between that of turkey origin and heterologous reovirus strains. The μNS gene was apparently very conserved, but 2016 and 2009 Hungarian TRVs clustered together and formed a common branch with two US origin TRVs, MN3 and 22342/13. All other US TRV strains formed a separate cluster in the μNS phylogenetic tree.

**Figure 4:**
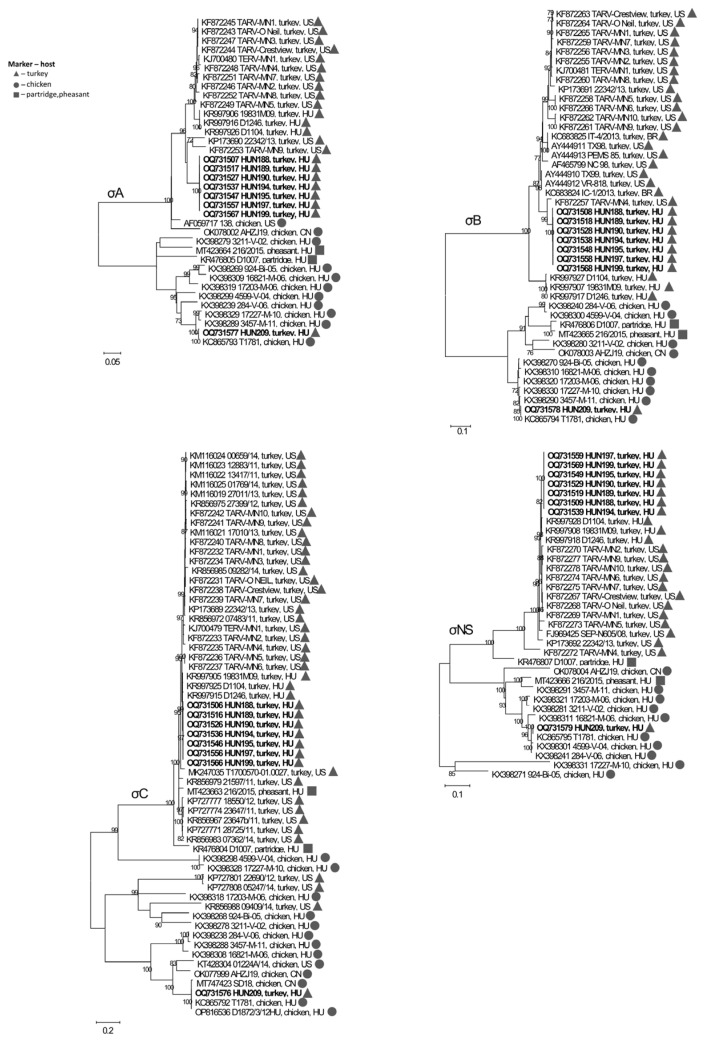
Unrooted phylogenetic trees based on the nucleotide sequences of the S-class genes of different ARVs. Markers indicate host species: triangle, turkey; circle, chicken; square, pheasant/partridge. The scale bar is proportional to the genetic distance. Bootstrap values greater than 60 are shown at the branch nodes.

The σA-based phylogenetic tree showed a similar topology for the TRV sequences with those described for λA and μA (Figure 4). Regarding the σB gene, all 2016 Hungarian TRVs formed a common cluster with US and Brazilian strains and separated from 2009 Hungarian strains. Finally, both σC and σNS were found to be highly conserved and no minor branches were identified. In the data set containing all currently known TRV genomes, TRVs were typically most closely related to each other and belonged to the same monophyletic group, despite the different sequence identity values.

Of interest, the unique HUN209 TRV isolate consistently clustered with a 2012 Hungarian chicken reovirus strain, T1781.

## 4. Discussion

The first data related to TRVs were published in the early 1980s [29,30]. It soon became evident that reovirus commonly occurs in turkey flocks [1,31]. However, traditionally, reovirus infection is not considered a high priority pathogen that would seriously affect the rearing or fattening of turkeys. Typically, both morbidity and mortality remain low in flocks affected by TRV infection unless other pathogens co-circulate in TRV-positive turkey farms [1,32,33,34]. In the 2010s, turkey reovirus infections were reported to re-emerge in the United States where 10–20% morbidity and up to 10% mortality rates were seen in several affected farms. Simultaneously, novel TRV strains were identified; these novel strains carried uncommon genotypes of the σC antigen [13,26,28]. The importance of these novel strains and their capacity to spread across farms and country borders is currently unknown.

In this study, we investigated the genetic features of TRV strains isolated in 2016 in Hungary and compared them to other strains to uncover the source of infections and the evolution of field strains in the absence of vaccine pressure. When compiling the sequence data for the present study, we realized that genome sequence information for TRV accumulates slowly in public databases. The majority of strains with available genome sequence data comes from the United States, while some genomes were reported from Hungary and a few partial gene sequences are available from Brazil and Croatia [13,16,35]. Even with the advancement of rapid genome amplification and sequencing methods, roughly a dozen TRV whole-genome sequences were available at the time when we started to prepare this paper. Studies published so far have indicated that turkey reoviruses separated from waterfowl origin orthoreoviruses, and they constitute in most genes distinct phylogenetic clades separated even from chicken origin orthoreoviruses [14,27,28,36,37]. Of interest, shared genes were observed when comparing turkey-origin reovirus strains with partridge- and pheasant-origin orthoreoviruses [10,38].

Reovirus genotyping is based on the features of the σC coding gene [25]. Traditionally, five genetic clusters were distinguished, but in some more recent papers, these clusters were renumbered while other authors proposed new genetic clusters [13,25,39,40,41,42]. In the original genotyping scheme, predominating TRVs were classified into cluster 2. Additional data suggest that TRVs belonging to different clusters may also circulate in turkey flocks and our study adds further data to this finding. In this study, we isolated and characterized a cluster 3 TRV. Evidence shows that strains belonging to different clusters represent different antigenic types or serotypes [39]. Within the main TRV genotype (cluster 2), US TRVs were reported to represent a single common antigenic variant that is distinguishable from chicken-origin reoviruses [13]. Although we did not perform cross-neutralization in this study, the diversity within this gene of Hungarian strains was very low, suggesting shared antigenic features of the main neutralization antigen, σC. The lack of substantial diversification within the neutralization antigen is a bit surprising considering the marked genetic and antigenic variation seen in the σC of chicken-origin reoviruses [43,44] and raises questions concerning the distinguishing features between the evolution of this gene in the two closely related avian hosts, chickens and turkeys. One important difference that may significantly affect viral evolution is the systematic use of reovirus vaccines in chickens to prevent tenosynovitis. This practice dates back several decades ago in chickens, while vaccination, other than using autovaccines, is practically missing for TRVs [45]. Additionally, since σC is the cell binding protein and determines tissue tropism [46], a lack of diversification may suggest the need to preserve the conserved structure of the receptor binding antigen. Assuming that evolutionary constraints against diversification within σC is important, these phenomena may act synergistically. Extending the time window of TRV strain monitoring will likely shed light on this question and hopefully help resolve these slightly controversial findings.

In addition to σC, all but one gene had comparable diversity values (range, 0.02 to 0.07). Yet, gene-wise phylogenetic analyses suggested that TRV strains may undergo reassortment. Of interest, these reassortment events occur in the context of a genetically stable pool of genes. One apparent exception to this scenario was seen in the μB gene. This was the only gene where genetic interaction between chicken and turkey reoviruses was evident. Accordingly, an interesting question descending from this exception was whether reassortment between chicken and turkey reoviruses is restricted to this single gene. It also seems important to understand the mode, frequency and place of genetic interactions between heterologous reoviruses in the turkey rearing process. Because μB is localized in the outer capsid of the reovirus particle [47], one may speculate that it may influence either the antigenic features and consequently virus neutralization in vivo or it is involved in TRV biology in some other ways (e.g., virion maturation, transcriptase activation and cell entry [48]). Theoretically, these features could help this turkey-specific virus clade to survive in its natural host in the field. In general, it is not surprising that μB shows this marked diversity in TRVs, given that reoviruses of chicken also tend to exchange this gene readily even with heterologous reoviruses [9].

From a molecular epidemiological standpoint, the Hungarian TRV strains detected in two study periods, 2009 and 2016, seemed to cluster according to temporal patterns whereas the geographic origin of the isolates seemed to be less influential in the phylogenies. Based on our current knowledge of virus evolution, these findings are more likely to suggest clonal spread of TRVs in Hungary in the respective study period. Some genes were shared with reoviruses of other avian species, suggesting that some sort of gene flow among reoviruses of different host species may also occur, but no evidence was seen for the field circulation of TRV or for subsequent interaction among strains identified in Hungary. If this hypothesis is correct, then the complex reassortment pattern seen among the 2009 and the 2016 Hungarian strains as well as the US TRV strains involving the conserved gene pool should have occurred at locations other than those where the diseases of turkeys were diagnosed.

## 5. Conclusions

TRVs in Hungary were selected for genomic analysis several years apart, in 2009 and 2016. The study strains appeared genetically similar to each other in the respective study year, regardless of the location of the sampling site. Despite the limited sample size, the results of these studies indicate a common origin for TRVs that co-circulate in selected Hungarian turkey flocks. At present, the exact source where these strains disperse from is unclear. However, turkey chicks can be purchased only from a few commercial rearing facilities, and a focus on these facilities from the perspective of epidemiologic surveillance and disease prevention seems to be justified. Collecting samples and performing virus genome analysis from other countries where the meat-turkey industry is relevant would permit insight into the epidemiology of TRVs in Europe and uncover additional elements of the transmission chain of pathogenic TRVs.

## Figures and Tables

**Table 1:** List of SNVs observed in the coding sequences of study strains.

Gene	Sample ID	Position (nt)	AA Change
λB	195	1176	-
λC	188	1513	D > Y
	189	2964	-
	190	3473	L > P
	189	3488	D > G
μA	188	1554	-
μB	188	1522	S > A
	188	1626	-
	188	1896	-
μNS	188	602	L > S
	188	748	L > I
σB	188	825	-
σNS	194	180	-

## Data Availability

Sequence data were deposited in GenBank (OQ731500-OQ731579).
